# Hyaluronidase induces a transcapillary pressure gradient and improves the distribution and uptake of liposomal doxorubicin (Caelyx™) in human osteosarcoma xenografts

**DOI:** 10.1038/sj.bjc.6602626

**Published:** 2005-06-07

**Authors:** L Eikenes, M Tari, I Tufto, ØS Bruland, C de Lange Davies

**Affiliations:** 1 Department of Physics, The Norwegian University of Science and Technology, Høgskoleringen 5, 7491 Trondheim, Norway; 2 Department of Radiotherapy and Oncology, University of Oslo, The Norwegian Radium Hospital, Montebello, 0310 Oslo, Norway

**Keywords:** hyaluronidase, interstitial fluid pressure, microvascular pressure, liposomal doxorubicin, tissue distribution

## Abstract

Liposomal drug delivery enhances the tumour selective localisation and may improve the uptake compared to free drug. However, the drug distribution within the tumour tissue may still be heterogeneous. Degradation of the extracellular matrix is assumed to improve the uptake and penetration of drugs. The effect of the ECM-degrading enzyme hyaluronidase on interstitial fluid pressure and microvascular pressure were measured in human osteosarcoma xenografts by the wick-in-needle and micropipette technique, respectively. The tumour uptake and distribution of liposomal doxorubicin were studied on tumour sections by confocal laser scanning microscopy. The drugs were injected i.v. 1 h after the hyaluronidase pretreatment. Intratumoral injection of hyaluronidase reduced interstitial fluid pressure in a nonlinear dose-dependent manner. Maximum interstitial fluid pressure reduction of approximately 50% was found after injection of 1500 U hyaluronidase. Neither intratumoral nor i.v. injection of hyaluronidase induced any changes in the microvascular pressure. Thus, hyaluronidase induced a transcapillary pressure gradient, resulting in a four-fold increase in the tumour uptake and improving the distribution of the liposomal doxorubicin. Hyaluronidase reduces a major barrier for drug delivery by inducing a transcapillary pressure gradient, and administration of hyaluronidase adjuvant with liposomal doxorubicin may thus improve the therapeutic outcome.

Liposomal doxorubicin may enhance the antitumour effect, and reduce the toxicity by improved pharmacokinetics compared with free doxorubicin. The polyethylene glycol (pegylated) coating reduces cellular reticuloendothelial system uptake and prolongs the circulation time (Gabizon *et al*, 1994). This, in combination with the ability of liposomes to extravasate through leaky tumour vessels (Wu *et al*, 1993; Yuan *et al*, 1994), contributes to selective localisation of liposomal doxorubicin in tumour tissue (Gabizon and Martin, 1997; Symon *et al*, 1999). In normal tissue, the intact liposomes are confined to the intravascular space, as normal blood vessels are not fenestrated to the same degree as in tumour tissue. Hence, the toxicity to normal tissue is reduced considerably, especially cardiotoxicity (Harris *et al*, 2002). Although liposomal delivery of doxorubicin improves the tumour uptake compared to free doxorubicin, the drug distribution within the tumour tissue is still heterogeneous (Vaage *et al*, 1997; Davies *et al*, 2004).

The enzyme hyaluronidase degrading hyaluronan is reported, in phase I and II trials, to prevent regrowth of various tumour types and to improve clinical outcome when given adjuvant to chemotherapy (Baumgartner, 1987; Maier and Baumgartner, 1989; Smith *et al*, 1997), and delays the growth of human breast cancer xenografts compared with doxorubicin given alone, when given as a pretreatment to doxorubicin (Beckenlehner *et al*, 1992). However, it has been reported that such pretreatment may induce drug resistance (Chang, 2001). Experiences from various clinical trials show that hyaluronidase seemingly has no toxicity towards normal tissues or and other adverse effects when used in various clinical trials (Bruera *et al*, 1999; Menon *et al*, 1999; Albanell and Baselga, 2000). This is also shown in preclinical experiments (Shuster *et al*, 2002). The mechanism for the enhanced therapeutic effect is not well known, but degradation of the ECM is assumed to improve the penetration of the drug. Delivery of drug to tumour cells occurs by two independent mechanisms: diffusion due to the concentration gradient and convection due to pressure gradients. In avascular multicellular spheroids, diffusion is the only mechanism of transportation, and the observed improved penetration of doxorubicin into spheroids incubated with hyaluronidase is thus probably due to this mechanism (Kohno *et al*, 1994). The high interstitial fluid pressure (IFP), which is driven by the microvascular pressure (MVP), is a major obstacle to penetration of therapeutic molecules, as the transcapillary pressure gradient is low, and an outward interstitial flux is generated toward the periphery of the tumour due to the steep pressure gradient in the periphery of the tumour (Boucher and Jain, 1992; Eikenes *et al*, 2004). We have previously shown that hyaluronidase reduced IFP (Brekken *et al*, 2000a) and periodic changes in IFP by hyaluronidase increased the tumour uptake of radiolabelled moab in human osteosarcoma xenografts (Brekken *et al*, 2000b).

Based on the above, we postulated that hyaluronidase might induce a transcapillary pressure gradient, thereby improving the tumour uptake and distribution of liposomal doxorubicin within the tumour. To test this hypothesis, IFP and MVP were measured by the wick-in-needle and micropipette technique, respectively, after intratumoral or intravenous injection of hyaluronidase in athymic mice bearing human osteosarcoma xenografts. The tumour uptake and distribution of liposomal doxorubicin injected i.v. 1 h after the hyaluronidase injection was studied on tumour sections by confocal laser scanning microscopy (CLSM).

## MATERIAL AND METHODS

### Animal and tumour models

Xenografts were grown in 7–9-week-old athymic female BALB/c nu/nu mice (Taconic, M&M, Denmark) either orthotopically (o.t.) or subcutaneously (s.c.), by injecting 100 *μ*l suspension of 2 × 10^6^ human osteosarcoma cells from the cell line OHS (Fodstad *et al*, 1986) either adjacent to the periosteum of the femurs or subcutaneously. The orthotopic tumours were growing around and infiltrating the bones. Growth kinetics and histology of the two tumour models have been reported previously (Brekken *et al*, 2000a). The xenografts were grown for 3–6 weeks, and the tumour sizes ranged from 0.7 to 1.7 cm^3^. The animals were kept under pathogen-free conditions and were allowed food and water *ad libitum*. All animal experiments have been carried out with ethical committee approval. The ethical guidelines that were followed meet the standards required by the UKCCCR guidelines.

### Treatments

The mice were anaesthetised with an s.c. injection of Hypnorm/Dormicum/sterile water,1 : 1 : 2, 4 ml kg^− 1^ bodyweight (Janssen Pharmaceutical, Belgium, and Alpharma AS, Norway) during all surgical and experimental procedures. Hyaluronidase (Neopermease®, Sandoz-Novartis, kindly provided by Professor G Baumgartner) was injected intratumorally in various concentrations diluted in PBS to a final volume of 50 *μ*l, or 50 *μ*l (1500 U in 50 *μ*l) was administrated into the tail vein. Liposomal doxorubicin (Caelyx™, Schering Plough, Norway) was injected into the tail vein 1 h after hyaluronidase administration, using 16 mg kg^− 1^ in 200 *μ*l PBS.

### Interstitial fluid pressure

Interstitial fluid pressure was measured in orthotopic and subcutaneous tumours using the wick-in-needle technique (Fadnes *et al*, 1977). In brief, a hypodermic needle (size G23) was placed centrally in the tumour and connected to a pressure transducer (SensoNor 840, Norway) via polyethylene tubing (PI-50, Becton-Dickinson, MD, USA) filled with sterile heparinised PBS (70 U ml^− 1^). Pressures were monitored online using a MacLab analogue to digital recording system with a sampling rate of 4 Hz (MacLab4/e, ADInstruments, UK). Fluid communication between the needle and the tumour tissue was tested by compression and decompression of the polyethylene tubing and accepted when the IFP did not differ more than 20%.

Prior to needle insertion, the pressure was set to zero at the equator of the tumour. The needle was carefully inserted into the centre of the tumour mass, allowing the IFP to equilibrate for 10–15 min. After reaching a stable IFP value, hyaluronidase was injected. Interstitial fluid pressure was recorded continuously up to 100 min after the injection. Control tumours received PBS.

### Microvascular pressure measurements

Microvascular pressure was measured in subcutaneous tumours using micropipettes and a servo-controlled counter-pressure system (Wiederhielm *et al*, 1964). The counter-pressure was generated to balance the change in the electrical resistance in the micropipette. The generated counter-pressure represents the actual MVP and was amplified and recorded using the same MacLab analogue to digital recording system as for the WIN technique. A micromanipulator (Narishige MMN-333, Japan) and a stereomicroscope (Nikon SMZ-10A, Japan) were used to place the pipette into visible, peripheral vessels. The micropipettes were made from borosilicat glass capillaries (GC100-15, 1.0 mm o.d. × 0.58 mm i.d., Harvard Apparatus, UK) using a horizontal pipette puller (PN-3, Narishige, Japan). A microbeveller (TMB 100 Turbo Microbeveller, WPI, UK) grounded the tip of the micropipettes, making a tip diameter of about 5 *μ*m.

The skin overlying the tumour was removed before micropuncture, and zero pressure was recorded in the saline film covering the wound. Microvascular pressure was measured in one vessel per tumour. After the first MVP measurement, 50 *μ*l hyaluronidase (1500 U) was injected intratumorally or i.v. The micropipette was withdrawn during the injection to avoid breakage of the pipette. Control tumours received 50 *μ*l PBS alone, given intratumourally or i.v. Microvascular pressure was measured up to 100 min after the hyaluronidase injection, but was not measured continuously throughout the experiment due to replacement of the pipette because of frequent pipette breakage and clogging.

The MVP was only measured in s.c. tumours because at this tumour site it was possible to observe the microvessels after removing the overlying skin. The IFP was also measured in o.t. tumours as this is physiologically a more correct mode.

### Preparation of tumour sections and immunofluorescence staining

Mice were killed by cervical dislocation 2 days after injection of liposomal doxorubicin, the tumours were excised, embedded in tissue Tec (O.C.T.™ Coumpound, Sakura, Netherlands), frozen in liquid N_2_ and 5 *μ*m thick tumour sections were mounted on glass slides.

When staining blood vessels, the tumour sections were fixed in acetone for 10 min and incubated in goat serum to prevent nonspecific binding. The vessels were stained using rat anti-mouse CD31 antibody (clone MEC13.3; PharMingen, CA, USA) (16.7 *μ*g ml^− 1^) for 1 h, followed by either biotinylated goat anti-rat IgG (Vector Lab, CA, USA) (10 *μ*g ml^− 1^) and streptavidin Alexa Fluor 633 (Molecular Probes, OR, USA) (100 *μ*g ml^− 1^), when colocalised with doxorubicin, or by rabbit anti-rat IgG (diluted 1 : 200) (DakoCytomation, Glostrup, Denmark), goat anti-rabbit Dako Envision (100 *μ*l) (DakoCytomation, Glostrup, Denmark) and rabbit anti-goat IgG Alexa Fluor 488 (10 *μ*g ml^− 1^) (Molecular Probes, Eugene, OR, USA), when colocalised with hyaluronan.

When staining hyaluronan, the tumour sections were incubated with PBS containing 3% bovine serum albumin for 1 h to prevent nonspecific binding. Hyaluronan was labelled by incubating the sections for 1 h with biotinylated hyaluronan-binding protein (b-HABP, Seikagaku America, MA, USA) (10 *μ*g ml^− 1^), followed by incubation with stuptavidin Alexa Fluor 633 (Molecular Probes, OR, USA)(10 *μ*g ml^− 1^) for 30 min.

When staining DNA, the tumour sections were equilibrated briefly in PBS and incubated with Hoechst 33258 (Molecular Probes, OR, USA) (1 *μ*g ml^− 1^) for 5 min and rinsed in PBS for 1 min.

All incubations were carried out at room temperature in a humid chamber, and the sections were rinsed with PBS after each incubation.

### Confocal laser scanning microscopy

Released doxorubicin, blood vessels, hyaluronan and DNA were localised on frozen tumour sections using a confocal laser scanning microscope objective (LSM510, Zeiss, Germany). Colocalisation of doxorubicin and blood vessels was examined using an X20/0.3 objective. The colocalisation of doxorubicin and DNA was studied using an X63/1.2 water objective. The colocalisation of hyaluronan, DNA and blood vessels was studied using an X40/1.2 water objective. A 488 nm argon laser line was used to excite doxorubicin and rabbit anti-goat-IgG-Alexa488, a 633 nm HeNe laser line was used to excite streptavidin Alexa Fluor 633 and a two-photon mode locked Ti : sapphire laser (Mira Model 900 – F, Coherent, Inc., Laser Group, Santa Clara, CA, USA) at *λ*=780 nm was used to excite Hoechst 33258.

Doxorubicin encapsulated in liposomes had a concentration that quenched the fluorescence. Doxorubicin does not bind covalently to the cells and was therefore washed away during the vessel labelling procedure. Colocalisation of doxorubicin and blood vessels had, therefore, to be assessed by sequentially imaging. The doxorubicin distribution was recorded before labelling the vessels, followed by imaging the same section and position with stained vessels. The sections were imaged along a radial track from the periphery to the centre. The start position of the track was marked, and based on this mark as well as the vessel structures the two images were easily overlayed. All images had a resolution of 512 × 512 pixels. Laser current and detector gain were chosen to minimise noise and to utilise all grey values.

The uptake of doxorubicin was quantified by measuring the average fluorescence intensity per image. The fluorescence intensity was measured along a horizontal and vertical radial track from the periphery through the centre to the other periphery. In all, 6–16 images were recorded per section depending on the position of the section within the tumour and the size of the tumour. This was carried out for 10 sections per tumour and three tumours per group of treatment. Each section was 150–200 *μ*m apart, and the first section was approximately 2 mm from the tumour periphery. Quantitative measurements require that the fluorescence is not attenuated nor bleached. As the sections were only 5 *μ*m thick, attenuation will negligible. Bleaching of doxorubicin fluorescence was measured by scanning sections continuously for 2 min. No change in the fluorescence intensity was observed, demonstrating the absence of bleaching with the settings used.

### Statistical analysis

Curve fitting of theoretical models to experimental data was performed using Sigma Plot (SPSS Inc. Headquarters, IL, USA). The changes in pressures, relative to pretreatment values, were determined individually for each tumour, and then averaged. Gaussian pressure data were analysed using Student's *t* tests (Minitab, Minitab Inc., State College, PA, USA).

Statistical analysis of doxorubicin fluorescence intensity data at each time point was performed using one-way analysis of variance, ANOVA. The frequency of images as a function of fluorescence intensity was analysed using the 5 × 2 contingency table and Chi-squared test. The contigency table contained a number of images with intensity values of 0–5 for the two groups, Caelyx™ alone or combined with hyaluronidase. All statistical analysis was performed using the significance criterion of *P*=0.05.

## RESULTS

### Effect of hyaluronidase on IFP

**Figure 1:**
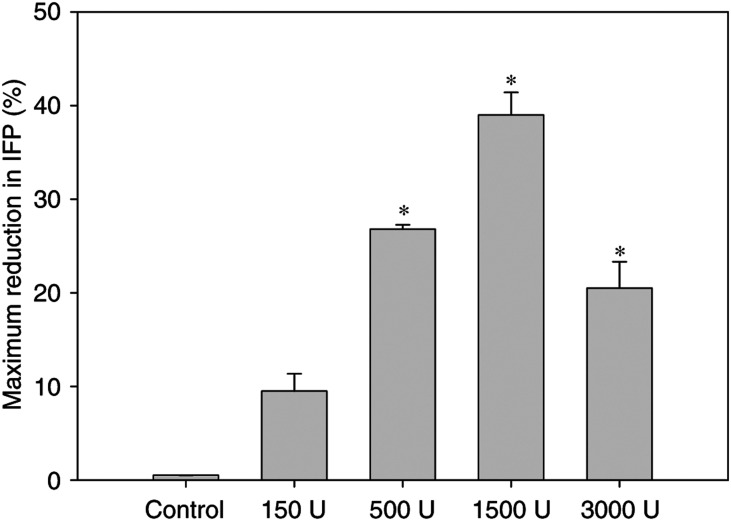
Maximum reduction (%) in IFP 1 h after intratumoral injection of PBS (*n*=5), 150 U (*n*=6), 500 U (*n*=5), 1500 U (*n*=6) and 3000 U (*n*=6) hyaluronidase in o.t. tumours. Each value is the mean of *n* tumours, the bars represent s.e. ^*^Data significantly different from untreated controls.

**Figure 2:**
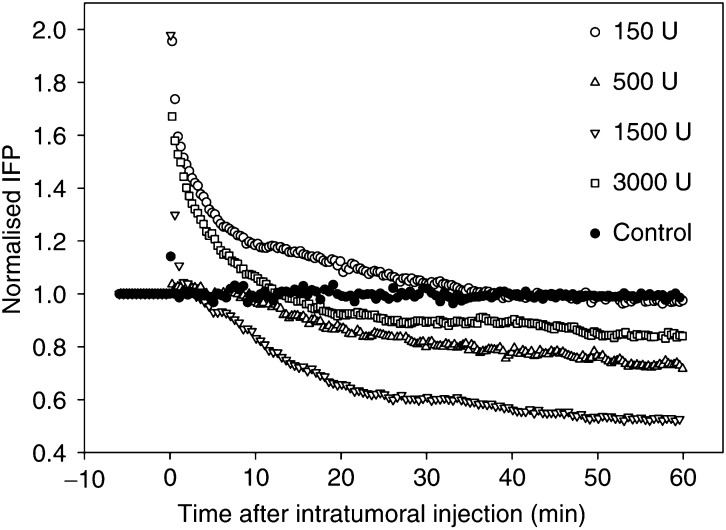
Interstitial fluid pressure response as a function of time after intratumoral injection of 150 U () (*n*=4), 500 U (Δ) (*n*=5), 1500 U (∇)(*n*=4) and 3000 U (□) (*n*=6) hyaluronidase in o.t. tumours. Interstitial fluid pressure is normalised to IFP before the injection. Each curve is the mean of *n* measurements.

Intratumoral injection of hyaluronidase in o.t. tumours reduced IFP in a dose-dependent manner up to a maximum reduction of approximately 40% after 1500 U of hyaluronidase (*n*=6) (Figures 1, 2). However, by increasing the dose further to 3000 U (*n*=6), IFP was reduced to a lesser extent; approximately 20% (Figure 1). A further increase to 5000 U was lethal. Statistical analysis showed that for all doses of hyaluronidase except 150 U (*n*=6) (*P*=0.052), the maximum reduction in IFP was significantly lower than the constant IFP level measured in control tumours injected with PBS (*P*<0.05). In addition, the reduction induced by 1500 U of hyaluronidase was significantly greater than the reduction induced by the other doses (*P*<0.05).

Maximum reduction in IFP was obtained approximately 1 h after hyaluronidase injection. The time response of the IFP reduction after intratumoral injection of hyaluronidase was biexponential with a fast (*τ*_v_) and a slow (*τ*_i_) time constant (Figure 2). The time response was normalised to the pressure before the hyaluronidase injection. An initial increase in IFP was observed, probably due to an increase in the volume and compression of the tissue at the moment of the intratumoral injection. Fitting the experimental data to the theoretical biexponential function 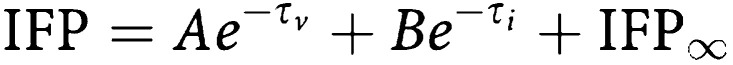 gave the time constants representing the IFP reduction. The time constants were approximately *τ*_v_≈50 s and *τ*_i_≈10 000 s, respectively, for the doses up to 1500 U. Hyaluronidase (3000 U) induced a slower reduction with time constants of approximately *τ*_v_≈300 s and *τ*_i_≈20 000 s.

**Figure 3:**
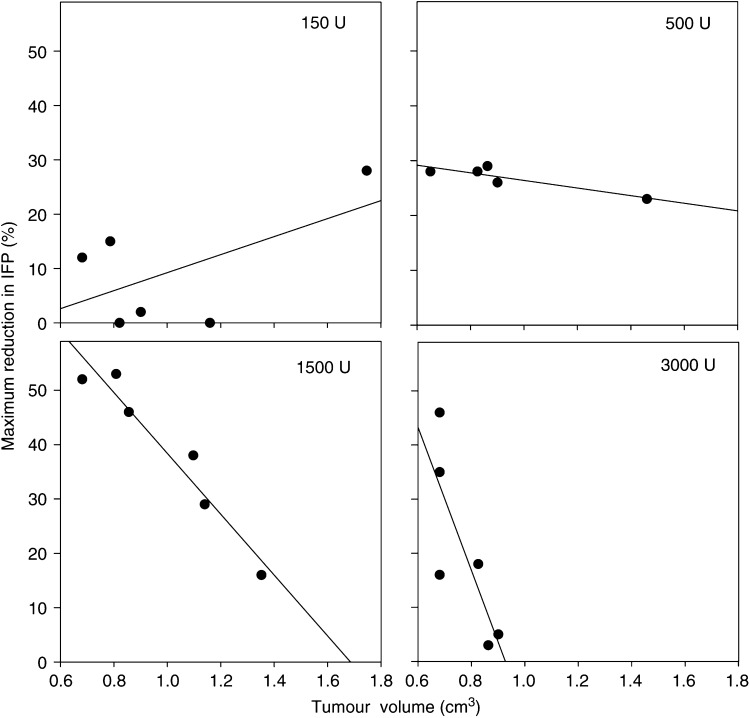
Maximum reduction in IFP after intratumoral injection of hyaluronidase as a function of tumour volume. Each symbol represents one measurement, the solid line represents linear regression analysis and was found to be significant for all doses except 150 U.

The maximum reduction in IFP correlated inversely with the tumour volume (Figure 3), except for the lowest dose used (150 U). Statistical analysis showed a significant correlation for 500 U (*R*^2^=0.79, *P*=0.044) and for 1500 U (*R*^2^=0.97, *P*=0.00), and almost significant for 3000 U (*R*^2^=0.64, *P*=0.055). Interstitial fluid pressure was thus reduced to a greater extent in the smaller tumours.

**Figure 4:**
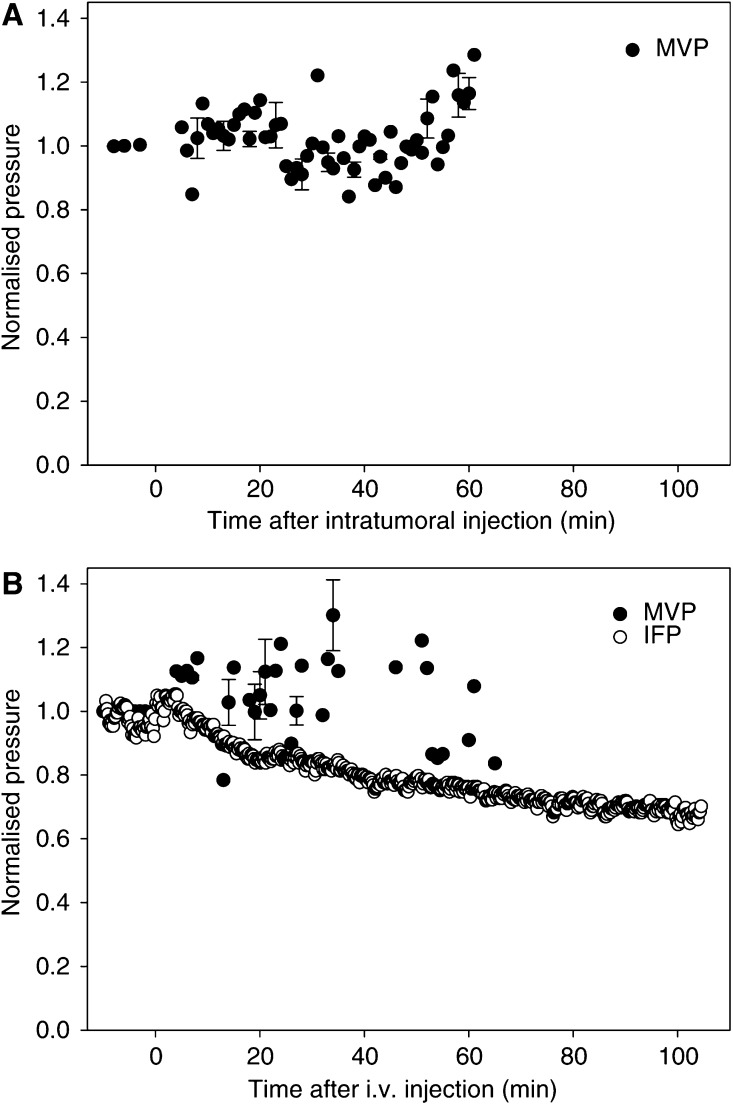
Normalised pressure after (**A**) intratumoral and (**B**) i.v. injection of 1500 U hyaluronidase in s.c. tumours. Microvascular pressure (•) was not measured continuously, and the data are based on different time intervals for *n*=5 (intratumoral injection) and *n*=7 (i.v. injection) mice. The bars represent s.e. at some time points. The IFP (○) graph is the mean of *n*=2 measurements.

Intravenous administration of 1500 U hyaluronidase in s.c. (*n*=2) tumours induced approximately the same maximum reduction in IFP as the intratumoral injection of 1500 U hyaluronidase (Figure 4B). However, the time response was longer, and the maximum reduction stabilised 100 min after the hyaluronidase injection.

### Effect of hyaluronidase on MVP

Intratumoral (*n*=5) as well as i.v. (*n*=7) injection of neither 1500 U hyaluronidase nor PBS (data not shown) had any significant effect on MVP in s.c. tumours (Figure 4). Previous studies have shown that intratumoral injection of hyaluronidase does not affect mean arterial blood pressure either (Brekken *et al*, 2000a).

### Effect of hyaluronidase on hyaluronan in the tumour tissue

**Figure 5:**
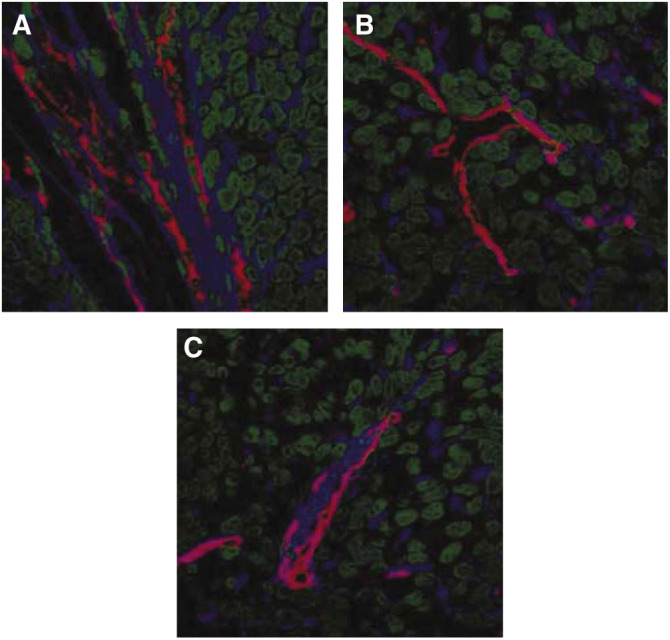
Tumour tissue hyaluronan (blue) in o.t. osteosarcoma xenografts relative to capillaries (red) and tumour cells (green) treated with PBS (**A**) and hyaluronidase (1500 U) (**B**, **C**).

Immunofluorescence staining showed that hyaluronan surrounded most of the vasculature in the untreated tumours. In addition, polymers of hyaluronan were located heterogeneously throughout the tumour (Figure 5A). Intense hyaluronan staining was seen at the rim of the tumours. Hyaluronidase had a diverse effect on the structure of hyaluronan, disintegrating both vascular associated and interstitial hyaluronan in some areas (Figure 5B), and leaving hyaluronan intact in other areas (Figure 5C). However, quanititative measurements of the hyaluronan fluorescence intensity showed no change after the hyaluronidase treatment (data not shown).

### Distribution of doxorubicin

**Figure 6:**
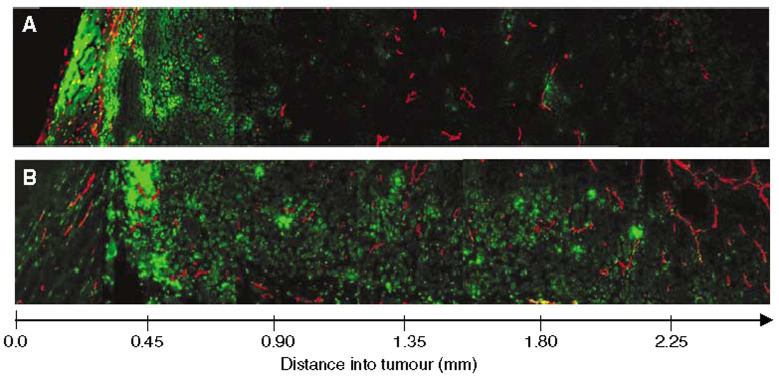
Distribution of liposomal doxorubicin in osteosarcoma xenografts treated with liposomal doxorubicin alone (16 mg kg^− 1^) (**A**) or liposomal doxorubicin combined with hyaluronidase (1500 U) (**B**). Representative images of doxorubicin (green) relative to capillaries (red) are presented from the rim to the centre of the tumour sections.

The distribution of doxorubicin in the periphery and central parts of the tumour was compared by imaging tumour sections along a radial track from the periphery to the centre of the section. In untreated tumours, liposomal doxorubicin was mainly located in the periphery of the tumour. The drug was found up to 450 *μ*m from the rim of the tumour. In tumours only treated with liposomal doxorubicin, hardly any doxorubicin fluorescence was detected in the central part of the tumours (Figure 6A). Hyaluronidase improved the distribution of doxorubicin considerably, although doxorubicin was still rather heterogeneously distributed. Hyaluronidase allocated doxorubicin further away from the vessels in the periphery, and doxorubicin was located around vessels in the central part of the section where no doxorubicin was observed in tumours given liposomal doxorubicin alone (Figure 6B).

### Quantitative measurements of doxorubicin uptake

**Figure 7:**
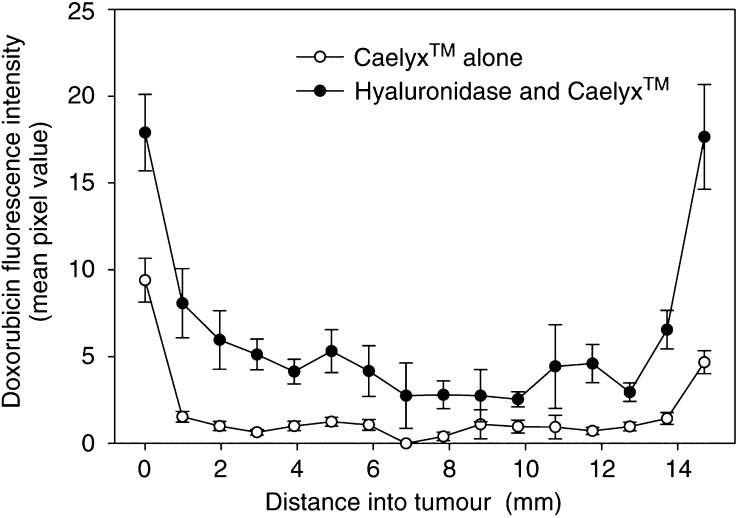
Doxorubicin fluorescence intensity profile in osteosarcoma xenografts treated with liposomal doxorubicin alone (16 mg kg^− 1^) (○) or combined with hyaluronidase (1500 U) (•). Each symbol is the mean of 5–20 images per section and 10 sections per tumour in three tumours. Bars represent s.e. The increase in the doxorubicin uptake in the hyaluronidase-treated tumours is significant throughout the whole tumour (*P*<0.05).

To obtain more quantitative data of doxorubicin distribution and uptake, the doxorubicin fluorescence intensity was quantified by estimating the average pixel intensity per image (Figure 7), and by estimating the fluorescence pixel area per image (data not shown). Both measurements gave similar results. The measurements were made along two perpendicular radial tracks on 10 sections per tumour in three tumours. The uptake of doxorubicin in the periphery was six to nine times higher than that in the other parts of the tumours when liposomal doxorubicin was given alone, and two to six times higher in the periphery of tumours treated with hyaluronidase (Figure 7). Hyaluronidase increased the uptake of doxorubicin two to eight times throughout the whole tumour, and in the central parts of the tumour hyaluronidase increased the uptake of doxorubicin approximately four times (Figure 7). An ANOVA comparing tumours treated with hyaluronidase and tumours given liposomal doxorubicin alone showed that the increase was significant throughout the whole tumour (*P*<0.05).

**Figure 8:**
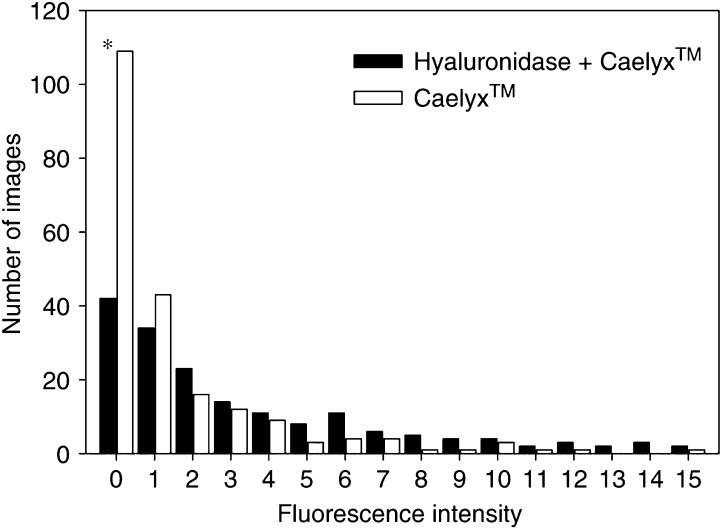
Number of images as a function of fluorescence intensity for osteosarcoma xenografts given liposomal doxorubicin alone (16 mg kg^− 1^) (□) or combined with hyaluronidase (1500 U) (▪). Each value is the mean of 5–20 images per section and 10 sections per tumour in three tumours. Bars represent s.e. ^*^Data significantly different from Caelyx™ alone.

The improved distribution of doxorubicin induced by hyaluronidase was demonstrated also by plotting the number of images as a function of fluorescence intensity. Tumours treated with liposomal doxorubicin and hyaluronidase had a higher number of images with detectable fluorescence than tumours treated with liposomal doxorubicin alone (Figure 8). A Chi-squared test showed that the number of images with no fluorescence was significantly higher for tumours that are only treated with Caelyx™. This shows that hyaluronidase redistributed doxorubicin to more areas of the tumours.

### Intracellular location of doxorubicin

**Figure 9:**
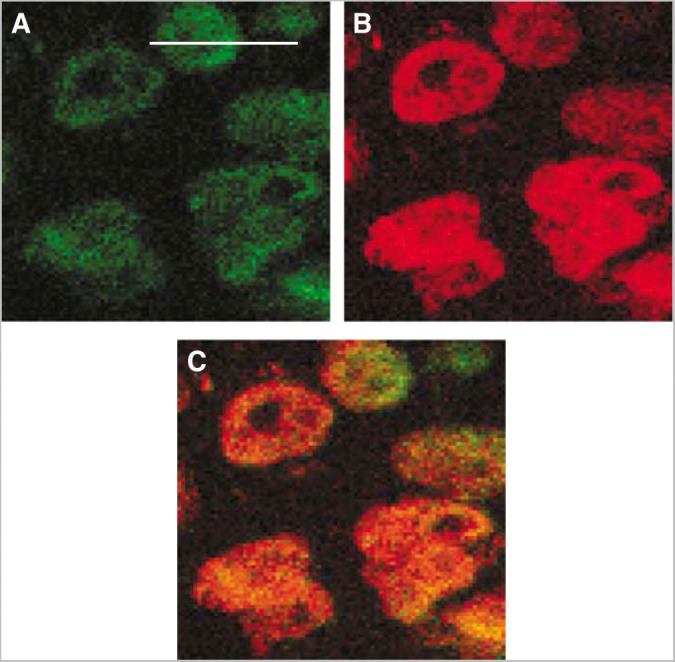
Intracellular distribution of doxorubicin (green) (**A**), DNA (red) (**B**) and colocalisation of doxorubicin and DNA (**C**) in osteosarcoma xenografts given liposomal doxorubicin alone (16 mg kg^− 1^). Bar=20 *μ*m.

Doxorubicin was located intracellularly, and hardly any fluorescence was seen in the ECM. The intracellular distribution of doxorubicin was mainly located in the nuclei, shown by colocalisation of doxorubicin and DNA (Figure 9). Hyaluronidase did not change the intracellular distribution.

## DISCUSSION

Doxorubicin is one of the most effective drugs in the management of osteosarcoma (Bruland and Pihl, 1997; Ferguson and Goorin, 2001). Although liposomal delivery of doxorubicin improves the tumour uptake compared to free doxorubicin, the drug distribution within the tumour tissue is still heterogeneous (Vaage *et al*, 1997; Davies *et al*, 2004). The present work showed that hyaluronidase increased the transcapillary pressure gradient in OHS xenografts, thereby improving the uptake and distribution of liposomal doxorubicin.

Hyaluronidase reduced IFP in a nonlinear dose-dependent manner, and the time-response of the IFP reduction followed a biexponential function. We have previously suggested that the fast initial time constant (*τ*_v_) reflects increased transcapillary transport, whereas the slower time constant (*τ*_i_) reflects interstitial transport (Brekken *et al*, 2000a; Eikenes *et al*, 2004). The present work demonstrated that the hyaluronidase-induced reduction in IFP was not caused by a reduction in MVP. Microvascular pressure remained constant and thus an increased transcapillary pressure gradient was established. Microvascular pressure is reported to govern IFP (Boucher and Jain, 1992) due to the lack of a lymphatic network and high vascular permeability. However, hyaluronidase reduced IFP independently of MVP, probably by the interstitial degradation of ECM. Hyaluronidase degrades extracellular hyaluronan by cleaving *β*-*N*-acetyl-hexoamine glycocidic bonds, which changes the overall structure of the ECM. Interstitial fluid pressure is reported to correlate inversely with the interstitial hydraulic conductivity (Jain and Baxter, 1988; Boucher *et al*, 1990), which correlates inversely with the concentration of glycosaminoglycans (Swabb *et al*, 1974). A reduction in glycosaminoglycans by hyaluronidase may thus increase the hydraulic conductivity, thereby reducing IFP. On the other hand, hyaluronidase induced a nonlinear reduction in IFP, and the interstitial degradation using the highest dose of hyaluronidase reduced IFP to a less extent. Higher doses of hyaluronidase may collapse the water swelling structure of hyaluronan to a greater extent, and induce crossbinding of the ECM fragments, making the ECM more viscous and less permeable. Such a nonlinear response has also been demonstrated when measuring the ability to penetrate the epithelial luminal glycocalyx after hyaluronidase treatment (Henry and Duling, 1999).

The reduction in IFP correlated inversely with tumour volume. This may be explained by a more efficient degradation of ECM due to the more homogeneous distribution of hyaluronidase in smaller tumours.

The route of administration of hyaluronidase had no impact on the observed pressure responses, neither IFP nor MVP. This is probably due to rapid vascular absorption of intratumoral-injected hyaluronidase, which has been demonstrated by a reduction in IFP in a secondary tumour only 40 s after an injection in the primary tumour (Brekken *et al*, 2000a).

Collagenase is another enzyme degrading the ECM. We have previously shown that i.v. administration of collagenase reduced IFP to the same extent and with the same kinetics as reported here for hyaluronidase (Eikenes *et al*, 2004). The IFP reduction was temporary and the recovery was similar for the two enzymes (Brekken *et al*, 2000a; Eikenes *et al*, 2004). Hyaluronidase did not induce any significant changes in MVP. Collagenase, however, reduced MVP, probably by reducing the vascular resistance as microvascular-associated collagen was disintegrated (Eikenes *et al*, 2004). Hyaluronan also surrounds vascular endothelial cells as an integrated part of the plasma membrane glycoproteins and glycolipids constituting the glycocalyx. The glycocalyx has been shown to reduce the vascular permeability (Qiao *et al*, 1995; Henry and Duling, 1999; Huxley and Williams, 2000; van Haaren *et al*, 2003) and contributes significantly to the hydraulic resistance of the capillary wall (Adamson, 1990). The glycocalyx also restricts the access of blood-borne macromolecules and cells to the luminal surface of the endothelial cells, thus reducing the functional diameter of the microvessels (Henry and Duling, 1999). Hyaluronidase has been found to increase the functional diameter for 70–145 kDa dextrans, but not for larger dextrans and red blood cells. However, the anatomic diameter was not changed (Henry and Duling, 1999); thus, hyaluronidase has probably no effect on the vascular resistance. Consistent with this, immunofluorescence staining of hyaluronan demonstrated that hyaluronidase induced a heterogeneous degradation of hyaluronan, and vascular-associated hyaluronan was only disintegrated around some vessels. Furthermore, quantitative measurements of the hyaluronan fluorescence intensity showed no change in the intensity of the hyaluronidase-treated sections, indicating that disintegrated hyaluronan is not removed and/or the treatment may increase the number of binding sites for the hyaluronan-binding protein as also found in a previous study (Brekken *et al*, 2000a). Other glycoproteins and proteoglycans as well as collagen also stabilise the vessel wall and contribute to the vascular resistance. Thus, the stability of the MVP after hyaluronidase treatment is probably due to the maintenance of the vascular resistance.

The distribution of liposomal doxorubicin in untreated tumours was heterogeneous, and mainly located in the periphery of the tumours. This correlates spatially with the peripheral IFP gradient previously measured in subcutaneous OHS xenografts (Eikenes *et al*, 2004), where the high central IFP decreased rapidly in the periphery of the tumour (0–400 *μ*m from the rim). In addition to high IFP, the poor distribution may be due to heterogeneous vascularisation and perfusion, slow interstitial diffusion and efficient extravascular binding of doxorubicin mainly in the tumour and endothelial cells.

Hyaluronidase increased the tumour uptake and improved the distribution of liposomal doxorubicin. Doxorubicin was located mainly around blood vessels in the periphery as well as in the central parts of the tumour, where hardly any doxorubicin was seen in untreated tumours. This suggests that the enhanced transcapillary pressure gradient increased the transcapillary transport of liposomes. Liposomes able to extravasate were probably degraded efficiently (Martin, 1997) as few intact liposomes were seen, and the released doxorubicin penetrated somewhat further into the ECM. Intact Caelyx™ liposomes have previously been observed to remain within the vasculature or are interstitial in very close proximity to the blood vessels (Davies *et al*, 2004), indicating that these large molecules are not able to penetrate away from the blood vessels and reach further into the interstitium.

Hyaluronidase may also increase the interstitial diffusion coefficient of doxorubicin. In avascular multicellular spheroids where diffusion is the only transport mechanism, hyaluronidase has been found to increase the penetration of doxorubicin (Kohno *et al*, 1994). On the other hand, in tumours growing in dorsal window chambers, both a fast and slow diffusive fraction of macromolecules were demonstrated, and i.v. injection of hyaluronidase increased the proportion of the slowly diffusing IgG and reduced the low diffusion coefficient, whereas no effect was seen for the fast diffusing IgG (Alexandrakis *et al*, 2004). A positive correlation between the diffusion coefficient of IgG and the amount of hyaluronan has also been reported (Davies *et al*, 2002). All together, this may reflect that the structure of the ECM is more important for the penetration of macromolecules than the amount of hyaluronan and other ECM constituents (Pluen *et al*, 2001; Brown *et al*, 2003).

Hyaluronidase seems to be more efficient than radiation in improving the distribution and uptake of Caelyx™ in osteosarcoma xenografts. We have previously reported that radiation (8 Gy) increased the uptake of Caelyx™ in the central part of the tumour two to four times (Davies *et al*, 2004). This suggests that the direct degradation of ECM is more efficient in improving the transport of macromolecules than in inactivating and inducing apoptosis in the tumour cells and thereby modulating the ECM.

The present work demonstrates that hyaluronidase reduces the barrier for drug delivery by inducing a transcapillary pressure gradient. An efficient transcapillary pressure gradient is especially important for the extravasation of large molecules such as liposomes. Pre- or coadministration of hyaluronidase adjuvant with liposomal doxorubicin may thus improve the therapeutic outcome.
